# Study of Janus Amphiphilic Graphene Oxide as a High-Performance Shale Inhibitor and Its Inhibition Mechanism

**DOI:** 10.3389/fchem.2020.00201

**Published:** 2020-04-15

**Authors:** Kaihe Lv, Pan Huang, Zhishi Zhou, Xin Wei, Qi Luo, Ziming Huang, Hui Yan, Han Jia

**Affiliations:** ^1^Key Laboratory of Unconventional Oil & Gas Development, China University of Petroleum (East China), Ministry of Education, Qingdao, China; ^2^Shandong Key Laboratory of Oilfield Chemistry, School of Petroleum Engineering, China University of Petroleum (East China), Qingdao, China; ^3^CNPC Tarim Oilfield Branch Oil and Gas Engineering Research Institute, Korla, China; ^4^School of Pharmacy, Liaocheng University, Liaocheng, China

**Keywords:** graphene oxide, Janus amphiphilic nano-sheets, shale inhibitor, plugging agent, inhibition mechanism

## Abstract

Janus amphiphilic graphene oxide (JAGO), modified by dodecylamine on one side of graphene oxide (GO), was investigated for its novel use as a shale inhibitor. JAGO was synthesized by the Pickering emulsion template technology and was characterized by the Fourier-transform infrared spectra, UV-vis spectra, and transmission electron microscopy. Compared to KCl (5%), polyether diamine (2%), and pristine GO (0.2%), JAGO's highest shale recovery rate (75.2% at 80°C) and lowest swelling height of Mt-pellets (2.55 mm, 0.2%) demonstrated its excellent inhibitive property. Furthermore, JAGO acted as a perfect plugging agent and greatly reduced filtration loss. Based on the results of X-ray diffraction, contact angle measurements, and pressure transmission tests, we proposed that the 2D nano-sheet amphiphilic structure of JAGO, which enabled it to be effective both in chemical inhibition and physical plugging, was responsible for its remarkable inhibition performances.

## Introduction

The global consumption of oil and gas has increased steadily in the past decades. In order to meet the enormous demand for energy, it has become necessary to exploit unconventional shale reservoirs (Mohr and Evans, 2010). Shale is mainly composed of clay materials, including montmorillonite, illite, illite/smectite formation, and kaolinite, and is extremely sensitive to water (Lishtvan et al., 2009; Labani and Rezaee, 2015). On contact with water, shale expands multiple times and disperses into the drilling fluids (Oort, 2003). The intensive chemical and mechanical interaction between shale and water can cause serious problems in drilling operations, such as stuck pipes, bit balling, tight holes, caving, and even loss of wells (Zeynali, 2012; Gholami et al., 2018; Lv et al., 2020). Therefore, proper selection of drilling fluids is vital in the exploitation of shale reservoirs. Oil-based drilling fluid is used in extreme cases; however, its high cost and the damage it causes to the environment restricts its application to some extent (Patel et al., 2007; Shivhare and Kuru, 2014). Researchers are focused on developing high-performance water-based drilling fluid (WBDF) by adding certain additives (shale inhibitors) to inhibit the swelling and hydration of shale.

A variety of chemicals have been used as shale inhibitors, including inorganic salts, ionic liquids, polymers, organic amines, and ammonium compounds (Shadizadeh et al., 2015; Barati et al., 2017; Jia et al., 2019a, 2020). All these chemicals are capable of inhibiting the swelling and hydration of shale to different degrees but show little ability in controlling the fluid loss in shale formations. As nanopores of shale have ultralow permeability, the fluid loss agent cannot pass through to form the filter cake (Zhang et al., 2008; Tang et al., 2014). Thus, water molecules can still enter the shale formation to weaken the effectiveness of shale inhibitors. To address the problem of water invasion, that is to reduce the filtration loss volume, many nanoparticles (NPs) have been used to plug the nanoscale pores and cracks in shale formation, such as nano-silica (Sensoy et al., 2009), aluminum salt (Liu et al., 2015), nano-emulsion (Xu et al., 2018a), and graphene (Aftab et al., 2016). Furthermore, much of the research has tended to focus on modified NPs that can largely reduce the swelling and hydration of shale through chemical interaction as well as physical plugging (Mao et al., 2015; Xu et al., 2018b; Zhong et al., 2018).

Graphene is the first two dimensional (2D) crystalline material with a single atom thickness, which was discovered by Geim and Novoselov (Novoselov, 2004). Graphene has a unique structure consisting of a single layer of carbon atoms, and it has been widely applied to various fields (Stoller et al., 2008; Xu et al., 2008; Wu et al., 2010). In the case of the drilling fluids industry, Aftab et al. (2016) reported that graphene could improve the rheological and filtration properties of WBDF at low temperatures and low-pressure conditions. Ridha et al. (2018) further proved the remarkable ability of graphene in filtration control at high temperatures. Moreover, An et al. (2016) demonstrated the high performance of ethylenediamine-modified graphene to plug nanopores and inhibit clay hydration. Previous investigations have indicated that graphene-based materials can effectively plug nanopores of shale to prevent water invasion. Thus, the inhibition of clay hydration could be achieved with modified graphene.

**Scheme 1:**
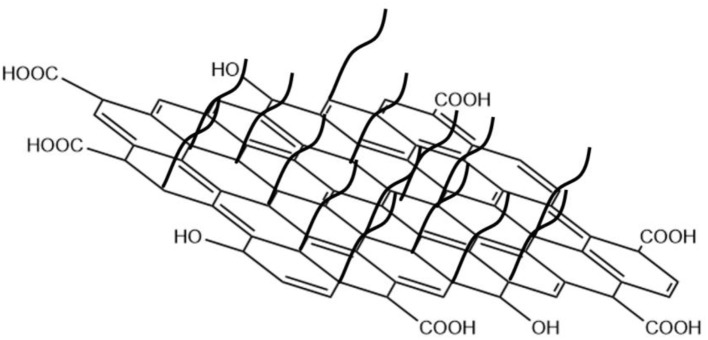
The structure of JAGO.

In this study, we investigate the potential application of Janus amphiphilic graphene oxide (JAGO, Scheme 1) as a shale inhibitor. JAGO refers to the graphene oxide nano-sheets that show hydrophilicity on one side and hydrophobicity on the other side, which is usually modified by an alkylamine (Wu et al., 2015). The amphiphilic property of JAGO enables its application in nanofluids as a flooding agent or emulsion stabilizer to enhance oil recovery (Luo et al., 2017; Chen et al., 2018). The inhibition and filtration control performance of JAGO was evaluated and compared with conventional inhibitors using laboratory experiments. The inhibition mechanism of JAGO was proposed based on the interaction analysis between JAGO and clay at the micro and macro scales.

## Experiment

### Materials

Dodecylamine (98%), paraffin wax (melting point ranges between 58 and 60°C), ethanol (97%), and silicon dioxide (99.5% 15 nm) were purchased from Aladdin Chemical Company, China. Chloroform (99%), KCl (99.8%), and NaCl (99.5%) were bought from Sinophram Chemical Reagent Co. Ltd., China. Graphene oxide was obtained from Turing Evolution Technical Company, China. Polyether diamine (PA) was provided by the Magcobar Mud Co. Ltd., China. Montmorillonite (Mt) and drilling fluid sodium bentonite (Na-bent) were purchased from the Weifang Huawei Company. The shale samples were provided by CNPC Chuanqing Drilling Engineering Company, which were obtained from the Weiyuan area, Sichuan Province, China. The shale compositions are listed in Table 1. Polytetrafluoroethylene film was the product of the Secco Experimental Equipment Company. All chemical reagents were used without further purification.

**Table 1:** Mineralogical composition of the shale samples.

**Component**	**Content (wt%)**	**Component of clay mineral**	**Relative content (wt%)**
Quartz	43.2	Illite/semctite	43.6
Calcite	4.4	Illite	32.6
Feldspar	6	Chlorite	13.6
Dolomite	7.4	Kaolinite	10.2
Clay mineral	39		

### Synthesis of JAGO

A detailed modification process of JAGO can be found in a report by Wu et al. (2015). A simplified description is as follows: First, graphene oxide (GO) was dispersed in deionized (DI) water at a concentration of 1 mg/mL with intensive sonication. Then a mixture of GO solution (200 mL), DI water (100 g), and paraffin wax (80 g) with 1% NaCl was heated to 75°C and stirred at 10,000 rpm with a FJ200-S homogenizer for 10 min. After cooling this down to an ambient temperature, the emulsion was filtered to obtain the GO-coated wax microspheres. Furthermore, the GO-coated wax was added to the ethanol solution of dodecylamine, and the dispersion was magnetically stirred for 12 h at 30°C. After washing with ethanol, the wax was dissolved by chloroform, following which, JAGO was obtained through centrifugation.

### Characteristics of JAGO

Fourier-transform infrared spectra (FTIR) analyses of GO and JAGO were conducted with a PerkinElmer Spectrum Two spectrometer (PerkinElmer, USA). UV–vis spectra of GO and JAGO were recorded by Cary 8454 UV-spectrophotometer (Agilent, USA). Transmission electron microscopy (TEM) images of GO and JAGO were obtained with a JEOL JEM-1400 transmission electron microscope (JEOL, Japan).

### Hot-Rolling Recovery Tests

Shale was crushed into small cuttings with sizes ranging between 2 and 5 mm. A certain amount of shale cuttings (M_1_) were added to a container filled with different inhibitor solutions. Then, the sealed container was rolled in a rolling oven at various temperatures for 16 h. After cooling it down to an ambient temperature, the remaining shale in the containers was washed with water and sieved through 40 mesh to get the recovered shale cuttings, which were weighted (M_2_) after being dried at 105°C for 4 h. Hot-rolling recovery rates were calculated using the following equation: A recovery = M_1_/M_2_.

### Linear Swelling Tests

Firstly, Mt-pellets were prepared by compressing 10 g Mt at 10 MPa for 10 min, and their initial heights were measured (H_0_). Then, the Mt-pellets were put in CPZ-2 expansion instruments (Qingdao, China), after which different inhibitor solutions at a certain concentration were poured into the expansion instruments. The Mt-pellets were found to expand with increasing time. The variation in height with respect to time was recorded. The final expansion rate was the value of the ultimate height divided by H_0_.

### Filtration Tests

A base slurry—which consisted of a dispersion of Na-bent in DI water, having a concentration of Na-bent at 4%, and Na_2_CO_3_ at 0.3%—was added to improve the hydration of Na-bent. After stirring for 2 h, the dispersion was subjected to pre-hydration for 24 h. Following this, different inhibitors were added at certain concentrations and static filtration tests were carried out following the API standard practice (APIRP 13B-1, 2009) at an ambient temperature and at a pressure of 0.69 MPa (APIRP 13B-1, 2009). The surface of the filter cakes was observed by scanning electron microscopy (SEM, JEOL JSM-6700F) after platinum sputter coating.

### Inhibition Mechanism Analysis

The hybrids of GO/Mt and JAGO/Mt were prepared according to the following procedure: First, 0.7 g of GO or JAGO was dissolved in 350 mL DI water by sonication. Then, a mass of 7 g Mt was added to the GO and JAGO solutions, respectively. After stirring for 2 h, the dispersion was centrifuged at 8,000 rpm for 10 min and was washed thrice with DI water to collect the precipitate.

A small part of the wet precipitate was used for X-ray diffraction (XRD) analysis (X'pert PRO MPD diffractometer, Netherlands). Then, the precipitate was dried at 105°C and ground to a powder for the XRD measurement of dry samples. The powder of GO/Mt and JAGO/Mt was compressed under 10 MPa for 10 min to make pellets. A contact angle goniometer (JC2000D5M, Zhongchen., China) was used to determine the wettability of the pellet surface.

Pressure transmission tests were conducted for various shale inhibitors to evaluate their physical plugging ability. The plugging fluids were the base slurry incorporated with different inhibitors. The procedure of this experiment was based on Huang et al. (2018). The experimental apparatus was the product of Jinzhou Modern Petroleum Science & Technology Co., Ltd.

## Results and Discussion

### Characterization of JAGO

**Figure 1:**
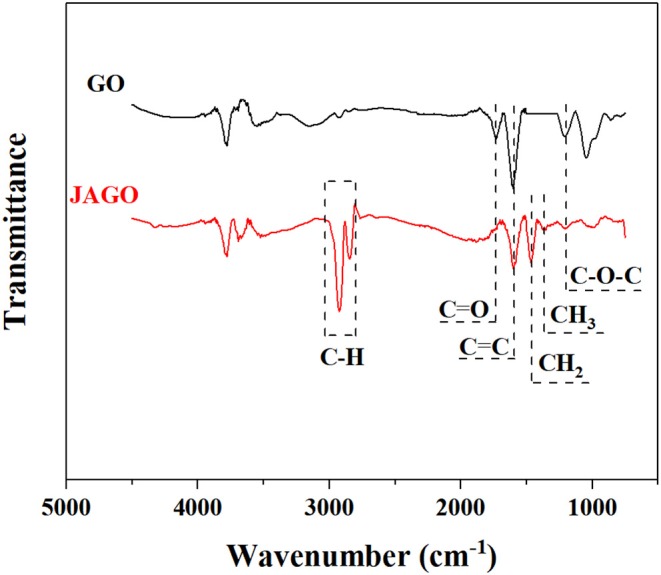
FTIR spectra of GO and JAGO.

**Figure 2:**
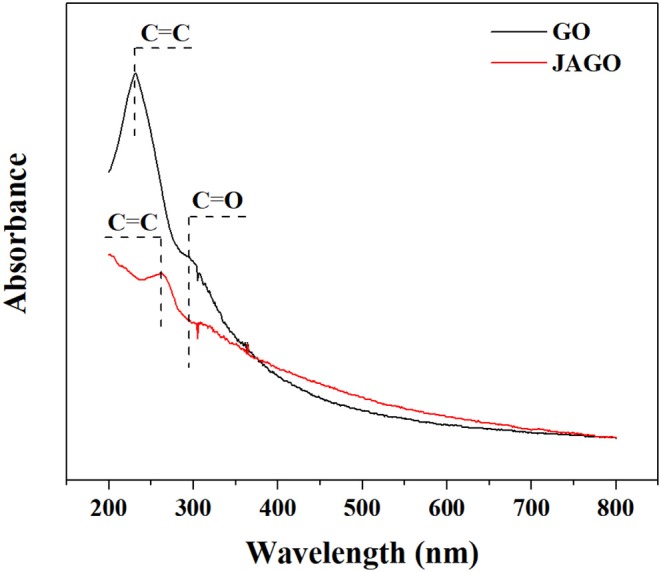
UV-vis spectra of GO and JAGO.

JAGO was modified according to the Pickering emulsion template technology reported by Wu et al. (2015). The successful modification was verified by the FTIR (Figure 1) and UV-vis spectra (Figure 2). As depicted in Figure 1, the spectrum pattern of JAGO presented distinguished differences to that of GO. Specifically, the intensive peaks (in the range of 3,100–2,800 cm^−1^), which were attributed to the C-H stretching vibration of the alkyl group as well as the weaker peaks at 1,463 and 1,380 cm^−1^, which were assigned to –CH_2_ and –CH_3_ (Acik et al., 2010; Huang et al., 2018), were observed in JAGO and not in GO. This was proof for the introduction of dodecylamine to GO. The weakened peaks at 1,728 and 1,225 cm^−1^, which referred to C=O and C–O–C, respectively, indicated the reaction sites on GO. The variation between UV-vis spectra of GO and JAGO (Figure 2) also supported the expected conjunction of dodecylamine onto GO. After modification, the peak at 302 nm, which was attributed to the n–π^*^ transitions of C=O vanished in the spectrum pattern of JAGO, and the peak standing for the π-π^*^ transitions of C=C shifted from 233 to 265 nm (Guardia et al., 2012). The increase in π-conjugation network could be responsible for the absorbance change (Choudhary et al., 2012).

**Figure 3:**
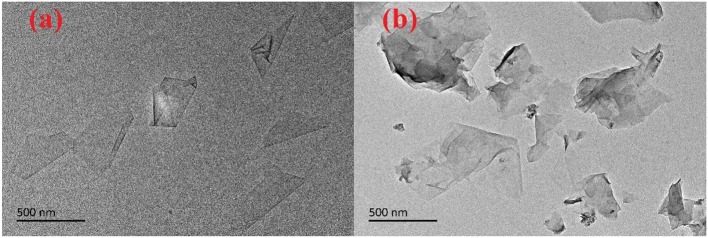
TEM images of GO **(a)** and JAGO **(b)**.

As shown in Figure 3, the TEM images of GO and JAGO presented a 2D structure. The lateral size was barely affected by the modification, whereas the conjunction of dodecylamine caused the overlap of JAGO to an extent (Figure 3b).

The amphiphilic property of JAGO was confirmed using a facile method. After shaking the mixture of the JAGO aqueous solution with oleic phase, such as octane, an interfacial film was formed at the oil/water interface (Figure S1). The generation of the interfacial film was attributed to the amphiphilic property of JAGO, which facilitated its interfacial adsorption and the construction of the interfacial film (Luo et al., 2016).

### Hot-Rolling Recovery Tests

**Figure 4:**
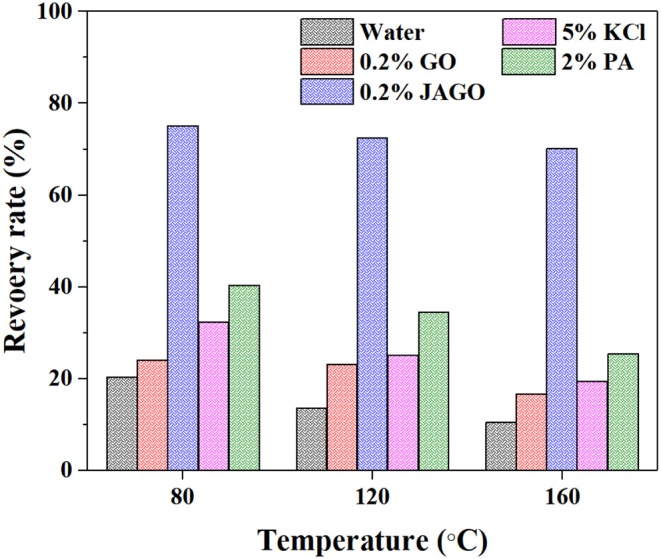
Hot-rolling recovery rates of shale cuttings in different inhibitor solutions at various temperature.

The hot-rolling recovery test is the most common method used to evaluate the inhibitive performances of the shale inhibitor. This method simulates the interaction between shale debris and water in drilling processes. Figure 4 presents the recovery rates of shale cuttings in different inhibitor solutions after rolling at 80, 120, and 160°C. The low recovery rates of shale in water (<20%) reflected the shale that was easily dispersed in water. However, the dispersion of shale was inhibited to different degrees with the addition of shale inhibitors. The pristine GO (0.2%) exhibited a weaker inhibitive capacity than PA (2%) and KCl (5%). The introduction of dodecylamine on one side of JAGO imbued it with an excellent inhibitive performance that was much better than found with GO and other conventional inhibitors. Seventy-five point two percentage of shale was recovered using JAGO (0.2%) at 80°C and the recovery rate was still over 70% at 160°C, which denoted the remarkable temperature tolerance of JAGO.

### Linear Swelling Tests

**Figure 5:**
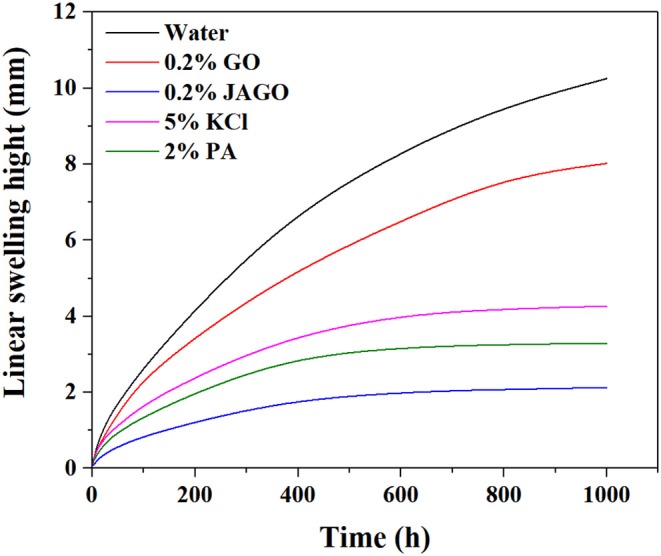
Linear swelling curves of Mt-pellets in different inhibitor solutions.

Linear swelling tests were also conducted to evaluate the inhibitive properties of JAGO. The final height of Mt-pellets in water, GO, JAGO, KCl, and PA solutions were 10.25, 8.02, 2.55, 4.27, and 3.29 mm, respectively (Figure 5), which yielded the corresponding reduction rates of 21.76, 75.12, 58.34, and 67.90%. Great improvement in the inhibitive performance was achieved by the asymmetric modification of JAGO with dodecylamine, which was owing to its 2D nano-sheet amphiphilic structure. The hydrophilic side of JAGO spontaneously adsorbed onto the hydrophilic surface of clay, while the hydrophobic side faced outward. Thus, a hydrophobic shield formed over the clay to prevent water from entering the interlayer of clay. A detailed mechanism analysis is given in the following sections.

### Filtration Tests

**Figure 6:**
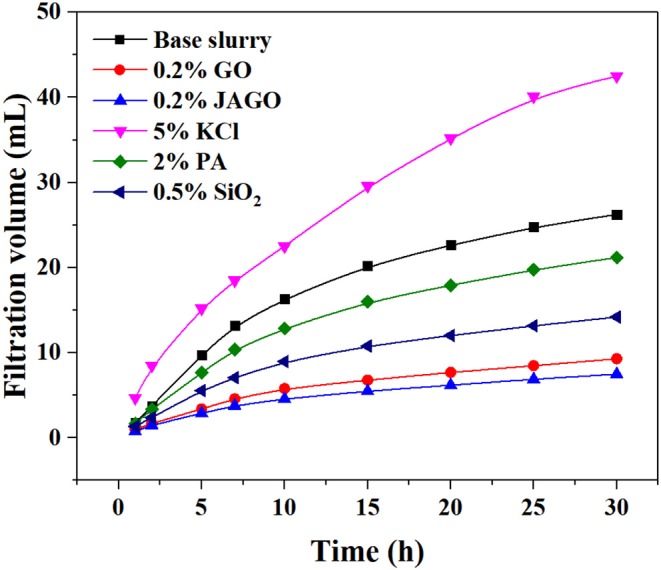
Filtration volumes of drilling fluids with the addition of various inhibitors.

**Figure 7:**
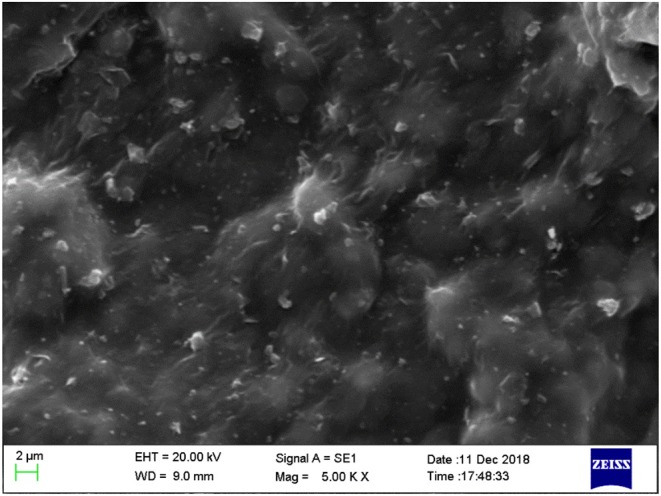
SEM image of the filter cake in the filtration test of JAGO.

Decreasing the filtration volume of drilling fluids is essential to reduce the hydration and swelling of shale. In order to evaluate the ability of JAGO to plug the nanopores in shale formation, we used the polyetrafluoroethylene membrane with nanoscale pores as the filter paper, instead of a normal paper with micropores. The filtration volume of the base slurry was 26 mL; moreover, the addition of GO and JAGO reduced the volume to 9 and 7 mL, which was much lower than PA (21 mL) and KCl (42 mL) (Figure 6). The performances of GO and JAGO were better than silica oxide (14 mL), the commonly used physical plugging agent. The SEM image of the filter cake revealed that the adsorption of JAGO onto the membrane fabricated the tight film (Figure 7), which sealed the nanopores in the shale formation to greatly prevent the invasion of drilling fluids. In addition, the hydrophobic exterior of the JAGO film on the membrane further impeded the invasion of water.

### Inhibitive Mechanism Analysis

#### XRD

**Figure 8:**
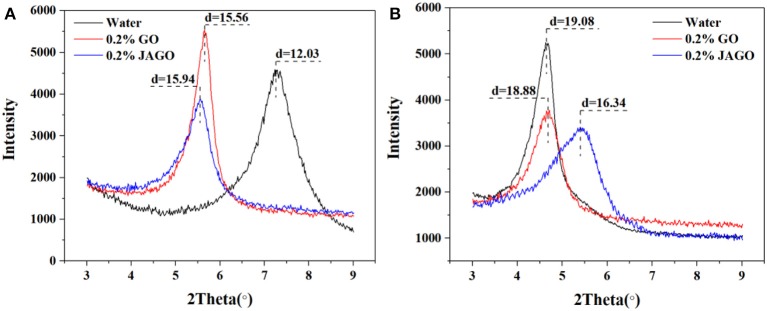
XRD patterns of Mt, Mt/GO and Mt/JAGO: dry samples **(A)**; wet samples **(B)**.

The XRD patterns of Mt, Mt/GO, and Mt/JAGO hybrids, including wet and dry samples are presented in Figure 8. For dry samples, the interlayer space (d_001_) of Mt, Mt/GO, and Mt/JAGO were 12.03, 15.56, and 15.94 Å, respectively. The increased value of d_001_ indicated that GO and JAGO successfully intercalated into the interlayer of Mt. In the case of wet samples, the interlayer space of the fully hydrated Mt was as large as 19.09 Å. After the intercalation of JAGO, the value of d_001_ was greatly reduced to 16.34 Å, whereas the intercalation of GO did not exert a significant effect on d_001_. The hydrophobic side of JAGO expelled water molecules from the interlayer in order to reduce the clay interlayer. The XRD results of the wet samples were in accordance with those in hot-rolling recovery tests and linear swelling tests.

### Contact Angle Measurement

**Figure 9:**
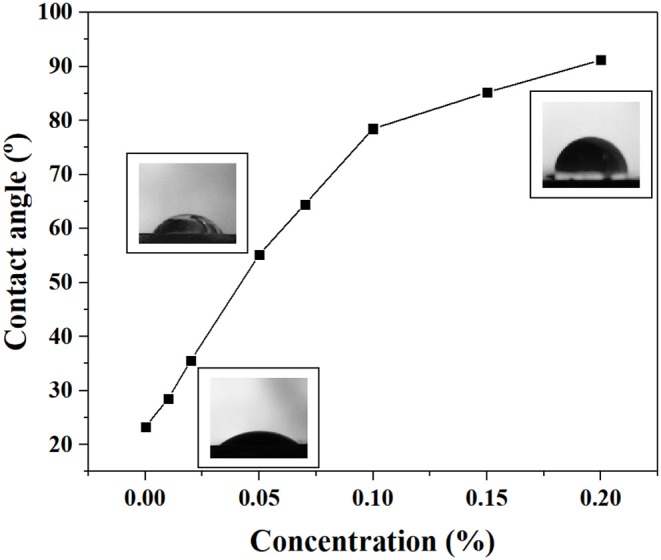
The contact angles of Mt after interacted with JAGO at different concentrations.

The contact angles of Mt interacting with JAGO are shown in Figure 9. With increasing concentrations of JAGO, the contact angle increased rapidly and dramatically, such that 0.2% JAGO could alter the Mt surface form strong water-wet to intermediate wettability with a contact angle of 91.2°. The wettability alteration verified our previous speculation that JAGO nano-sheets could adsorb onto the surface of clay with the hydrophobic side facing outward. The driving force of the adsorption derived from two aspects. On the one hand, the hydrophilic side of JAGO was rich in hydroxyl, carboxyl, and epoxy groups which could form hydrogen bonds with the oxygen atom and hydroxyl at the clay surface (McCoy et al., 2019). On the other hand, the hydrophobic side also facilitated the movement of JAGO toward the clay. Hence, JAGO adsorbed on the clay surface constructed a hydrophobic shield to effectively inhibit the hydration and swelling of clay.

### Pressure Transmission Tests

**Figure 10:**
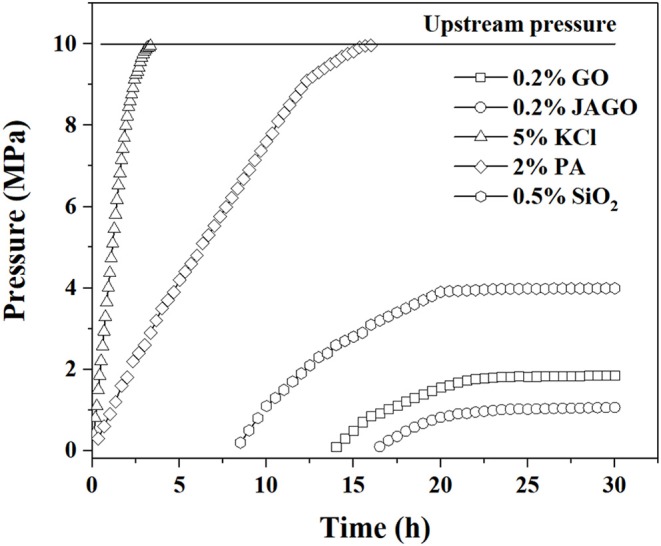
Pressure transmission tests of various plugging fluids.

The plugging ability of GO, JAGO, KCl, PA, and silica oxide were assessed by pressure transmission tests. The upstream pressure was fixed at 10 MPa and the downstream pressure was monitored by injecting plugging fluids. Variations in the downstream pressure compared to the injection time for different plugging fluids is exhibited in Figure 10. In the case of KCl and PA, the downstream pressure reached a constant value of upstream after 2.5 and 15 h, respectively, which indicated that KCl and PA had little plugging effects, leading to the penetration of fluids throughout the shale cores. Nevertheless, the pressure variation in the plugging fluids that contained nanoparticles displayed different tendencies. The downstream pressures of GO, JAGO, and SiO_2_ were at atmosphere pressure at the initial stage but began to increase after 14, 16.5, and 4.6 h, respectively. The ultimate downstream pressure of GO, JAGO, and SiO_2_ were 1.86, 1.07, and 3.99 MPa, respectively. The variation in downstream pressure demonstrated that JAGO was a perfect plugging agent, whereas GO was slightly weaker. Moreover, the aggregation of SiO_2_ might be responsible for its poor performance as it could degrade the property of SiO_2_ to plug nanopores.

### Inhibitive Mechanism of JAGO

**Figure 11:**
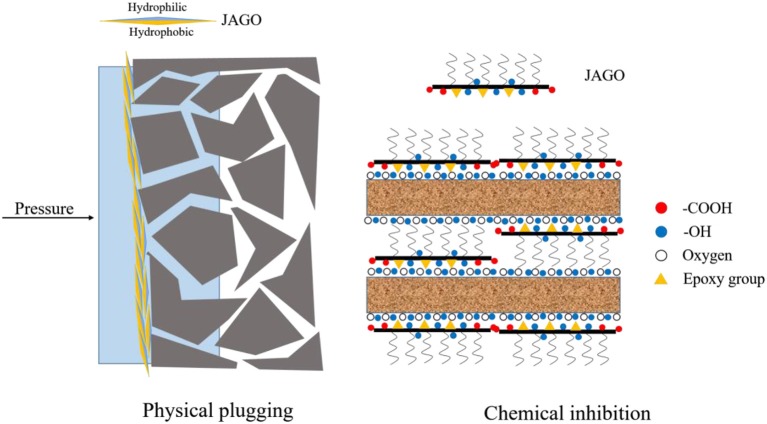
The proposed inhibitive mechanism of JAGO.

There are two essential mechanisms of shale inhibitors: chemical inhibition and physical plugging. Chemical inhibition is usually related to the chemical interaction of the inhibitor and clay, including adsorption, ion exchange, and intercalation into the interlayer space (Zaltoun and Berton, 1992; Jia et al., 2019b; Liu et al., 2019). The primary approach of physical plugging is plugging the pores, cracks, and natural flow channels to prevent drilling fluid invasion (Cai et al., 2012; Akhtamanesh et al., 2013). Based on the analysis of the above mechanisms, JAGO performed well in terms of chemical inhibition as well as physical plugging, as illustrated in Figure 11. Above all, due to the amphiphilic structure of JAGO, the hydrophilic side could spontaneously adsorb onto the clay surface, and the outward hydrophobic side formed a hydrophobic shield that impeded the interaction of clay and water. Furthermore, JAGO could intercalate into the interlayer space of clay to expel water molecules. Then, as the JAGO adsorbed onto shale, the 2D nano-sheets overlapped to construct a tight film on the shale, which perfectly sealed the nanopores in shale formation. The unmodified GO was a great physical plugging agent, whereas the chemical inhibition property, resulting from the conjunction of dodecylamine, enabled the JAGO to be an excellent shale inhibitor.

## Conclusion

This work innovatively introduced JAGO as a high-performance shale inhibitor that displayed both chemical inhibition and physical plugging properties. The inhibitive performance of JAGO was evaluated and compared with KCl (5%), PA (2%), and pristine GO (0.2%) by hot-rolling recovery tests and linear swelling tests, wherein JAGO (0.2%) achieved the highest recovery rate (75.2% at 80°C) and the lowest swelling height (2.55 mm). Chemical inhibition was attributed to the spontaneous adsorption of JAGO on a clay surface. Specifically, the hydrophilic side of JAGO adsorbed onto the clay surface via hydrogen bonds, and the hydrophobic side of JAGO faced outward. As a result, the fabricated hydrophobic shield greatly prevented water invasion, which was verified by contact angle measurements. In addition, the variation in clay interlayer spacing with and without JAGO indicated that JAGO intercalated into the interlayer of clay and expelled water molecules. In the case of the physical plugging property, JAGO reduced the filtration volume to 7 mL in the filtration tests and kept the downstream pressure at a relatively low value of 1.07 MPa with a constant upstream pressure at 10 MPa in the pressure transmission tests. The adsorbed 2D nano-sheet JAGO overlapped to construct a tight film, which effectively sealed the nanopores in shale formation. In summary, the difunctional JAGO, having excellent chemical inhibition and physical plugging properties, exhibited its potential application as a shale inhibitor. Nevertheless, further research is needed on issues such as the effect of JAGO on the rheological property of drilling fluids, its economic analysis, and different kinds of Janus amphiphilic nano-sheet shale inhibitors. Other 2D nano-sheet structure materials, including CuO sheet, TiO_2_ flakes, and certain nano-composites materials, could also be considered as shale inhibitors in future studies.

## Data Availability Statement

The raw data supporting the conclusions of this article will be made available by the authors, without undue reservation, to any qualified researcher.

## Author Contributions

KL was in charge of funding acquisition, conceptualization, and methodology. PH wrote the original draft. ZZ, XW, QL, and ZH conducted all the experiments and completed the data analyses. HY and HJ revised and edited the manuscript.

### Conflict of Interest

The authors declare that the research was conducted in the absence of any commercial or financial relationships that could be construed as a potential conflict of interest.
