# Supplementary material for: Study of Janus Amphiphilic Graphene Oxide as a High-Performance Shale Inhibitor and Its Inhibition Mechanism
Source: Front Chem. 2020 Apr 15;8:201. doi: 10.3389/fchem.2020.00201 (PMC7174724; doi:10.3389/fchem.2020.00201)
Supplement: Supplementary file 1 [file Data_Sheet_1.docx]

**Supporting Information**


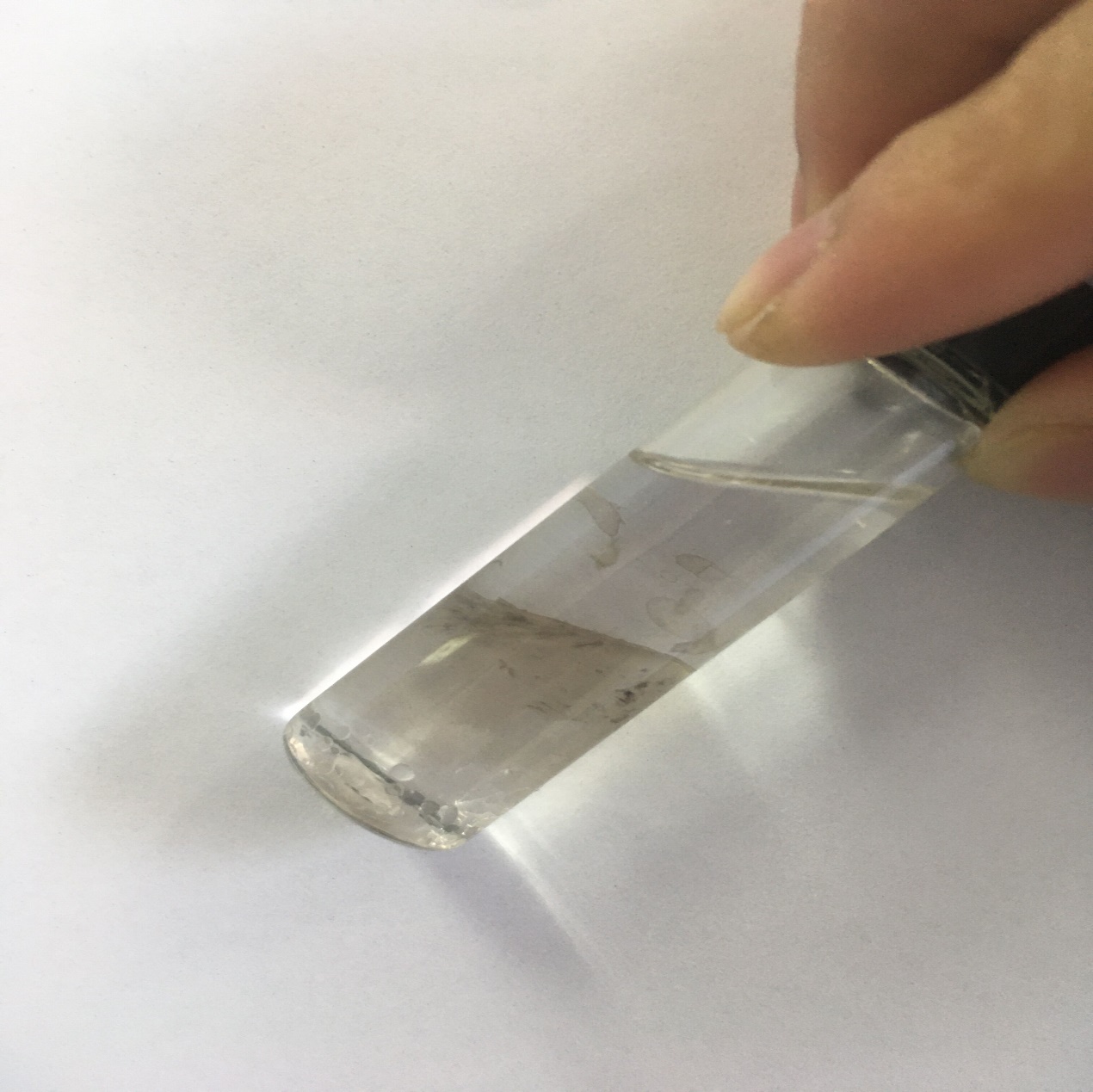


Figure S1. The JAGO interfacial film formed at the water/octane interface.
